# Two types of mental fatigue affect spontaneous oscillatory brain activities in different ways

**DOI:** 10.1186/1744-9081-9-2

**Published:** 2013-01-10

**Authors:** Yoshihito Shigihara, Masaaki Tanaka, Akira Ishii, Etsuko Kanai, Masami Funakura, Yasuyoshi Watanabe

**Affiliations:** 1 Department of Physiology, Osaka City University Graduate School of Medicine, 1-4-3 Asahimachi, Abeno-ku, Osaka City, Osaka, 545-8585, Japan; 2 Digital & Network Technology Development Center, Panasonic Co., Ltd, 1006 Kadoma, Kadoma City, Osaka, 571-8501, Japan; 3 RIKEN Center for Molecular Imaging Science, 6-7-3 Minatojima-minamimachi, Chuo-ku, Kobe City, Hyogo, 650-0047, Japan

**Keywords:** Magnetoencephalography (MEG), Mental fatigue, n-back test visual analogue scale (VAS)

## Abstract

**Background:**

Fatigue has a multi-factorial nature. We examined the effects of two types of mental fatigue on spontaneous oscillatory brain activity using magnetoencephalography (MEG).

**Methods:**

Participants were randomly assigned to two groups in a single-blinded, crossover fashion to perform two types of mental fatigue-inducing experiments. Each experiment consisted of a 30-min fatigue-inducing 0- or 2-back test session and two evaluation sessions performed just before and after the fatigue-inducing mental task session.

**Results:**

After the 0-back test, decreased alpha power was indicated in the right angular gyrus and increased levels in the left middle and superior temporal gyrus, left postcentral gyrus, right superior frontal gyrus, left inferior frontal gyrus, and right medial frontal gyrus. After the 2-back test, decreased alpha power was indicated in the right middle and superior frontal gyrus and increased levels in the left inferior parietal and superior parietal lobules, right parahippocampal gyrus, right uncus, left postcentral gyrus, left middle frontal gyrus, and right inferior frontal gyrus. For beta power, increased power following the 0-back test was indicated in the left middle temporal gyrus, left superior frontal gyrus, left cingulate gyrus, and left precentral gyrus. After the 2-back test, decreased power was suggested in the left superior frontal gyrus and increased levels in the left middle temporal gyrus and left inferior parietal lobule. Some of these brain regions might be associated with task performance during the fatigue-inducing trials.

**Conclusions:**

Two types of mental fatigue may produce different alterations of the spontaneous oscillatory MEG activities. Our findings would provide new perspectives on the neural mechanisms underlying mental fatigue.

## Background

Fatigue refers to the feeling that people may experience after or during prolonged periods of activity [1] and is a common problem in modern society [2]. Since fatigue decreases efficiency in the performance of daily activities, clarifying the mechanisms underlying fatigue and developing efficient methods for overcoming it would be beneficial. The neural mechanisms of mental fatigue are to some extent clarified: From the results of the studies using event-related potential, evaluation of predicted rewards and potential risks of actions were considered to be central to the phenomenon of mental fatigue and the evaluation system was considered to consist of a neural circuit that interconnects basal ganglia, amygdala, insular cortex, orbitofrontal cortex, prefrontal cortex, and anterior cingulate cortex [1]; in addition, the brain region associated with the sense of fatigue was identified as the orbitofrontal cortex [3].

Although definition of fatigue is not uniform, there seems to exist a common concept that fatigue is related to the impairment of performance rather than fatigue sensation [4-6]. Fatigue sensation is not fatigue itself, since fatigue sensation is involved in the biological alarm to order rest under the condition of fatigue; while fatigue itself is involved in the neural damage manifested as impaired performance [2]. Today, little is known about the neural mechanisms of mental fatigue related to the performance.

Mental fatigue is observed as a reduced efficiency for mental tasks [5]. Recently, new methods of induction and evaluation of mental fatigue have been proposed [7-9]. As a fatigue-inducing mental task session, participants performed 0- or 2-back test trials for 30 min. The 0-back test was used to represent a lower mental-load task, which could be performed without the use of working memory, while the 2-back test was used to represent a higher mental-load task, which required working memory [10]. The advantage of using these tasks is in their ability to cause two types of mental fatigue: After both 0-back and 2-back test sessions, impairment of task performance assessed by the percentage of correct task trials was significant, while longer reaction times and a higher subjective level of sleepiness could be identified only after the 0-back test trials. Since mental fatigue is multi-faceted [11], using two types of mental tasks might reveal different facets.

Although a variety of psychophysiological parameters have been used in previous research dealing with fatigue, electroencephalography (EEG) has been proposed as a promising indicator of mental fatigue [12]. The electrical activity of the brain is classified according to rhythms defined according to frequency bands, including beta, alpha, theta, and delta. Each frequency band is associated with specific information processing in the central nervous system [13]. Recently, we found that two types of mental fatigue affected spontaneous EEG alpha and beta power densities in different way [14]: After enrollment, 18 participants were randomly assigned to two groups in a single-blinded, crossover fashion to perform two types of mental fatigue-inducing experiments. Each experiment consisted of four 30-min fatigue-inducing 0- or 2-back test sessions and two evaluation sessions performed just before and after the fatigue-inducing sessions. Eleven electrodes were attached to the head skin, from positions F3, Fz, F4, C3, Cz, C4, P3, Pz, P4, O1, and O2. In the 2-back test, the beta power densities on the P3, Pz, and O1 electrodes and the alpha power densities on the P3, Pz, O1, and O2 electrodes were decreased, and the theta power densities on the Fz and Cz electrodes were increased after the fatigue-inducing mental task sessions. In the 0-back test, the alpha power densities on the P3 and Pz electrodes were decreased after the fatigue-inducing sessions. However, because of limited spatial resolution, it was impossible to identify the brain regions associated with the alteration of the EEG power densities. In addition, the roles of the EEG power changes caused by mental fatigue were unclear. Compared to EEG, magnetoencephalography (MEG) has the following advantages: (1) the magnetic field is hardly affected by intervening tissues; (2) measurements of the magnetic field do not require a reference; and (3) measurements from many sensors can be performed [15]. MEG shares with EEG the advantages of measuring brain activity by using time-frequency analyses and having a high temporal resolution. Therefore, alterations of spontaneous MEG alpha and beta power densities induced by mental fatigue may provide valuable clues to identify the neural mechanisms of fatigue. The aim of our study was thus to clarify how the neural underpinnings of mental fatigue relate to task performance using MEG focusing on the resting-state time-frequency analyses of the alpha and beta frequency bands.

## Methods

### Participants

Ten male healthy volunteers [30.8 ± 9.4 years of age (mean ± SD)] were enrolled in this study. They had normal or corrected-to-normal visual acuity, no history of medical illness, and were right-handed according to the Edinburgh handedness inventory [16]. The study protocol was approved by the Ethics Committee of Osaka City University, and all the participants gave written informed consent for participation in this study.

### Experimental design

**Figure 1:**
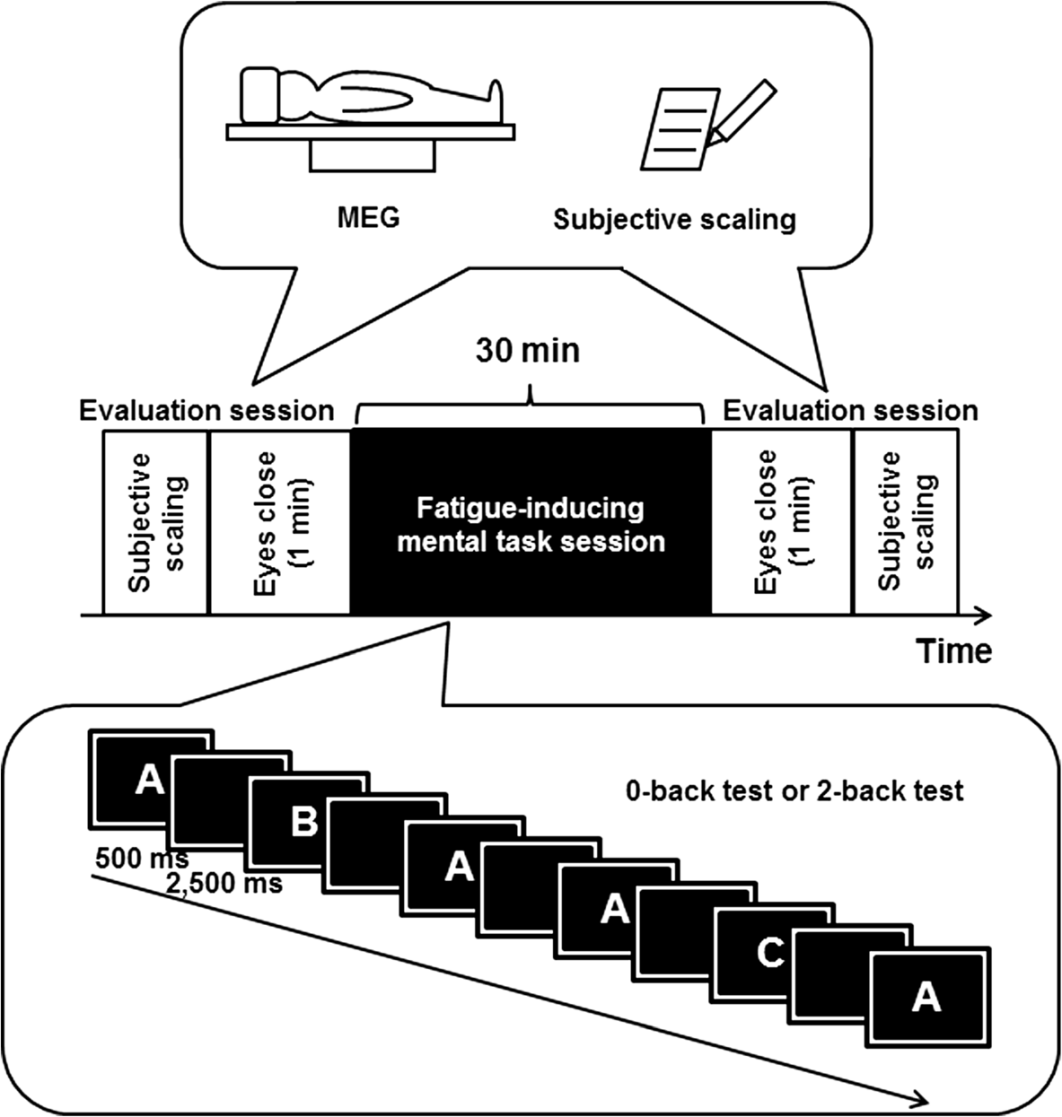
**Experimental design.** Each experiment consisted of one fatigue-inducing mental task session and two evaluation sessions performed just before and after the fatigue-inducing mental task session. During the fatigue-inducing mental task session, participants performed 0-back or 2-back test trials for 30 min. They were randomly assigned to two groups in a single-blinded, crossover fashion to perform the two types of the fatigue-inducing trials on separate days (the same participants performed both 0- and 2-back tasks and that order of task was counterbalanced, i.e., five participants began with the 0-back test and the others with the 2-back test). During the evaluation sessions, magnetoencephalography (MEG) recordings were performed with the participant’s eyes closed and subjective scaling.

This study was composed of two experiments in order to compare two types of mental fatigue. After enrollment, the participants were randomly assigned to two groups in a single-blinded, crossover fashion to perform two types of fatigue-inducing mental tasks (the same participants performed both 0- and 2-back tasks and that order of task was counterbalanced, i.e., five participants began with the 0-back test and the others with the 2-back test). An experiment was composed of three sessions: one fatigue-inducing mental task session and two evaluation sessions (Figure 1). During the fatigue-inducing mental task session, they performed 0-back or 2-back test trials for 30 min [10]. Just before and after the fatigue-inducing mental task session, MEG recordings were performed in the evaluation sessions with the subject’s eyes closed for 1 min in relaxed wakefulness, and subjective scaling using visual analogue scales (VAS’s) was performed before the first MEG recording and after the second recording. This study was conducted in a magnetically shielded room at Osaka City University Hospital. For 1 day before each visit, they refrained from intense mental and physical activities and caffeinated beverages, consumed a normal diet, and maintained normal sleeping hours. The healthy volunteers had not taken any form of medication during the last week. The time interval between the two experiments was 24 hr.

### Fatigue-inducing mental tasks

Participants performed a 0-back or 2-back test for 30 min on a bed of a MEG scanner lying in supine position during the fatigue-inducing mental task session. They watched a video projection screen located 30 cm straight ahead of their eyes. Any of four types of letters were continually presented on the video screen every 3 sec. The letter was presented on the screen for 500 ms and not presented for 2500 ms thereafter. During the 0-back trials, they were asked to press the right button with their right middle finger if the target letter (shown beside the video screen) was presented at the center of the video screen. If any other letters appeared, they were to press the left button with their right index finger. During the 2-back trials, they had to judge whether the target letter presented at the center of the video screen was the same as the one that had appeared 2 presentations before. If it was, they were to press the right button with their right middle finger, while if it was not they were to press the left button with their right index finger. They were instructed to perform the task trials as quickly and as correctly as possible. The result of each 0-back or 2-back trial - correct response or error - was continually presented on the display of the video screen. Performance on these tests was evaluated through the percentage correct and mean reaction time. To evaluate the time course of task performance during the fatigue-inducing mental task session, the performance measures were calculated every 5 min.

### Subjective scaling

Subjective scaling was performed to evaluate participants’ subjective levels of fatigue and sleepiness. The participants were verbally asked to rate their subjective levels of fatigue (in Japanese, tsukareteiru) and sleepiness (in Japanese, nemui) on a VAS from 0 (minimum) to 100 (maximum) using a paper-and pencil questionnaire [17].

### MEG recordings

MEG recordings were performed using a 160-channel whole-head type MEG system (MEG vision; Yokogawa Electric Corporation, Tokyo, Japan) with a magnetic field resolution of 4 fT/Hz^1/2^ in the white-noise region. The sensor and reference coils were gradiometers 15.5 mm in diameter and 50 mm in baseline, and each pair of sensor coils was separated at a distance of 23 mm. The sampling rate was 1,000 Hz with a 1 Hz high-pass filter.

### MEG data analyses

MEG signal data were analyzed offline after analogue-to-digital conversion. Epochs of the raw MEG data including artifacts such as running trains of subway were excluded from the analysis by careful visual detection of the artifacts before averaging. After averaging, the power was determined in two frequency bands, alpha (8–13 Hz) and beta (13–25 Hz) by a fast Fourier transformation using Frequency Trend (Yokogawa Electric Corporation) for each participant and the contrast images of (after the n-back test trials)/(before the n-back test trials) was constructed for each frequency band.

Anatomical magnetic resonance imaging (MRI) was performed using a Philips Achieva 3.0TX (Royal Philips Electronics, Eindhoven, Netherland) to permit registration of magnetic source locations with their respective anatomical locations. Before MRI scanning, five adhesive markers (Medtronic Surgical Navigation Technologies Inc., Broomfield, CO) were attached to the skin of the participant’s head (the first and second ones were located at 10 mm in front of the left tragus and right tragus, the third at 35 mm above the nasion, and the fourth and fifth at 40 mm to the right and left of the third one). The MEG data were superimposed on MR images using information obtained from these markers and the MEG localization coils. Localization and intensity of the time-frequency power of the cortical activity were estimated using the software Brain Rhythmic Analysis for MEG (BRAM; Yokogawa Electric Corporation) [18], which used narrow-band adaptive spatial filtering technique. These data were then analyzed using Matlab (Mathworks, Sherbon, MA), implemented in statistical parametric mapping (SPM8, Wellcome Department of Cognitive Neurology, London, UK). The MEG anatomical/spatial parameters used to warp the volumetric data were transformed into the Montreal Neurological Institute (MNI) template of T1-weighed images [19] and applied to the MEG data. The anatomically normalized MEG data were filtered with a Gaussian kernel of 15 mm (full-width at half-maximum) in the x, y, and z axes. The oscillatory power for each frequency band after the n-back test trials relative to that before the n-back test trials was measured on a region-of-interest basis. The resulting set of voxel values for each comparison constituted a SPM of the t statistics (SPM{t}). The SPM{t} was transformed to the unit of normal distribution (SPM{Z}). The threshold for the SPM{Z} of individual analyses was set at *P* < 0.001 (uncorrected for multiple comparisons). The weighted sum of the parameters estimated in the individual analyses consisted of “contrast” images, which were used for the group analyses [20]. So that inferences could be made at a population level, individual data were summarized and incorporated into a random-effect model [20]. SPM{t} and SPM{Z} for the contrast images were created as described above. Significant signal changes for each contrast were assessed by means of t statistics on a voxel-by-voxel basis [20]. The threshold for the SPM{Z} of group analyses was set at *P* < 0.001 (uncorrected for multiple comparisons). Anatomical localizations of significant voxels within clusters were done using the Talairach Demon software [21] with the nearest gray matter option enabled. We captured the fiducial coils and tracked the head position before and after the MEG recording. The criterion for head movement exclusion was the movement more than one voxel size (5 × 5 × 5 mm).

### Statistical analyses

Data are presented as mean ± SD unless otherwise stated. Two-way analyses of variance (ANOVA’s) for repeated measures were performed to evaluate the significance of difference among VAS values. Paired *t*-test was used to evaluate the significance of differences between two conditions. Simple regression analyses were conducted to evaluate the relationships between task performances and MEG variables. All P values were two-tailed, and values less than 0.05 were considered to be statistically significant. Statistical analyses were performed using the SPSS 20.0 software package (SPSS, Chicago, IL).

## Results

**Figure 2:**
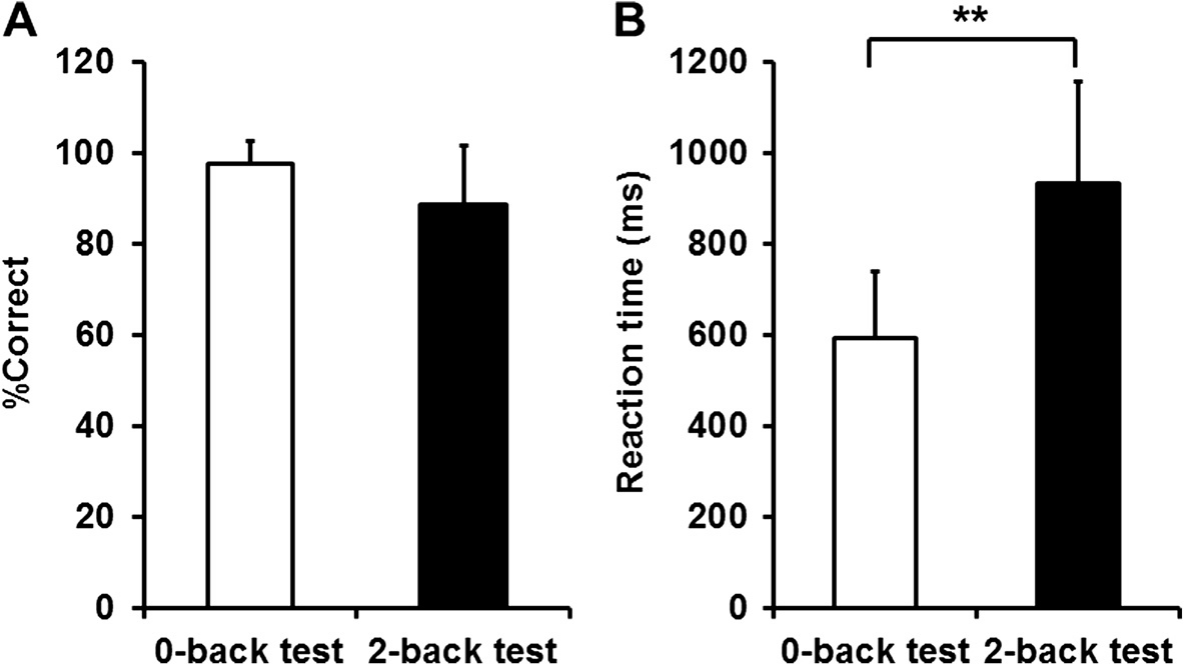
**Task performance during 0-back and 2-back test trials.** Rate of correct trials [% correct] (**A**) and reaction time (**B**) were assessed. Values are presented as the mean and SD. **P < 0.01, significant difference (paired *t*-test).

The task performance measures during 0-back and 2-back test trials are shown in Figure 2. Although the percentage correct of the 2-back test was not different from that of the 0-back test, the reaction time of the 2-back test was longer than that of the 0-back test.

The subjective level of fatigue was significantly affected by time course (pre- and post-task measurement) [F(1,9) = 18.05, P < 0.001], but not by task or by a task × time course interaction effect [F(1,9) = 0.09, P = 0.766]. No significant main effects were found for subjective level of sleepiness.

**Figure 3:**
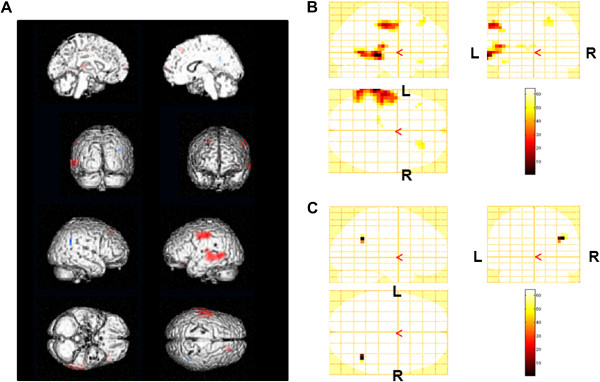
**Statistical parametric maps of alpha power changes by 0-back test trials (random-effect analyses of 10 participants, P < 0.001, uncorrected for the entire search volumes).** Statistical parametric maps are superimposed on surface-rendered high-resolution MRI’s, and red indicates an increase of alpha power and blue indicates a decrease (**A**). Increase of alpha power (**B**) and decrease of alpha power (**C**) were shown on sagittal (upper left), coronal (upper right), and axial (lower left) sections. Color bar (lower right) indicates % increase (**B**) or % decrease (**C**) of the alpha power. R, right; L, left.

Alpha power changes after the fatigue-inducing mental task sessions are shown in Table 1 and Figure 3. After the 0-back test, decreased power was shown in the right angular gyrus [Brodmann’s (BA) area 39] and increased levels were shown in the left middle temporal gyrus (BA 21), left superior temporal gyrus (BA 41), left postcentral gyrus (BAs 2 and 3), right superior frontal gyrus (BA 6), left inferior frontal gyrus (BA 47), and right medial frontal gyrus (BA 10). After the 2-back test, decreased power was shown in the right middle frontal gyrus (BAs 8 and 46) and right superior frontal gyrus (BAs 6 and 9) and increased levels were shown in the left inferior parietal lobule (BA 39), left superior parietal lobule (BA 7), right parahippocampal gyrus (BA 36), right uncus (BA 20), left postcentral gyrus (BA 3), left middle frontal gyrus (BA’s 6 and 10), and right inferior frontal gyrus (BA 40).

**Table 1:** Alpha power changes after fatigue-inducing task trials

	**Side**	**Brodmann’s area**	**Coordinates (mm)**			**Z value**
			**x**	**y**	**z**	
**0-back test**						
**Decrease**						
Angular gyrus	R	39	42	−52	30	3.23
**Increase**						
Middle temporal gyrus	L	21	−68	−32	−5	3.61
Middle temporal gyrus	L	21	−68	−52	0	3.43
Superior temporal gyrus	L	41	−48	−22	10	3.39
Postcentral gyrus	L	2	−58	−17	45	3.45
Postcentral gyrus	L	3	−53	−7	40	3.34
Superior frontal gyrus	R	6	22	38	55	3.18
Inferior frontal gyrus	L	47	−43	33	−15	3.15
Medial frontal gyrus	R	10	2	68	10	3.13
**2-back test**						
**Decrease**						
Middle frontal gyrus	R	8	52	28	40	3.69
Middle frontal gyrus	R	46	42	23	20	3.44
Superior frontal gyrus	R	6	17	43	55	3.40
Superior frontal gyrus	R	9	27	58	30	3.24
**Increase**						
Inferior parietal lobule	L	39	−53	−67	40	3.55
Superior parietal lobule	L	7	−33	−47	55	3.48
Parahippocampal gyrus	R	36	27	−27	−15	3.32
Uncus	R	20	32	−17	−40	3.24
Postcentral gyrus	L	3	−53	−12	55	3.18
Middle frontal gyrus	L	10	−28	53	−10	3.14
Middle frontal gyrus	L	6	−48	8	50	3.11
Inferior frontal gyrus	R	47	27	38	−10	3.10

x, y, z: Stereotaxic coordinates.

Random-effect analyses of 10 participants (*P* < 0.001, uncorrected for the entire search volumes).

**Figure 4:**
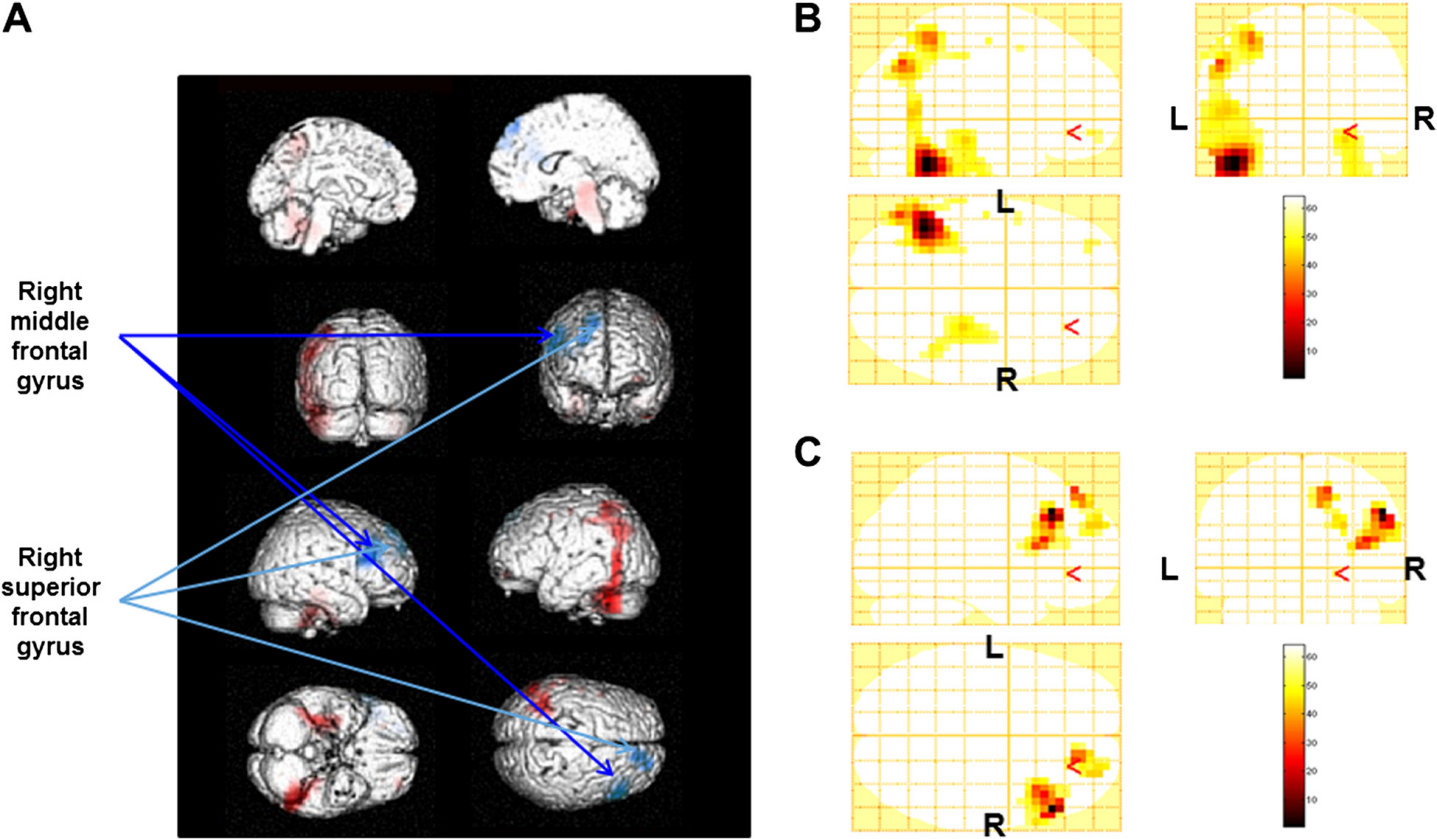
**Statistical parametric maps of alpha power changes by 2-back test trials (random-effect analyses of 10 participants, P < 0.001, uncorrected for the entire search volumes).** Statistical parametric maps are superimposed on surface-rendered high-resolution MRI’s, and red indicates an increase of alpha power and blue indicates a decrease (**A**). Increase of alpha power (**B**) and decrease of alpha power (**C**) were shown on sagittal (upper left), coronal (upper right), and axial (lower left) sections. Color bar (lower right) indicates % increase (**B**) or % decrease (**C**) of the alpha power. R, right; L, left.

Beta power changes after the fatigue-inducing mental task sessions are shown in Table 2 and Figure 4. After the 0-back test, increased levels were shown in the left middle temporal gyrus (BA 21), left superior frontal gyrus (BA 8), left cingulate gyrus (BA 31), and left precentral gyrus (BA 6). After the 2-back test, decreased power was shown in the left superior frontal gyrus (BA’s 6 and 9) and increased levels were shown in the left middle temporal gyrus (BA 39) and left inferior parietal lobule (BA 40).

**Table 2:** Beta power changes after fatigue-inducing task trials

**Location**	**Side**	**Brodmann’s area**	**Coordinates (mm)**			**Z value**
			**x**	**y**	**z**	
**0-back test**						
**Decrease**						
**Increase**						
Middle temporal gyrus	L	21	−63	−37	−5	3.89
Superior frontal gyrus	L	8	−13	43	50	3.45
Cingulate gyrus	L	31	−23	−42	20	3.30
Precentral gyrus	L	4, 6	−63	8	30	3.09
**2-back test**						
**Decrease**						
Superior frontal gyrus	L	6	−18	38	55	3.21
Superior frontal gyrus	L	9	−28	58	25	3.18
**Increase**						
Middle temporal gyrus	L	39	−63	−62	5	3.24
Inferior parietal lobule	L	40	−48	−52	35	3.18

x, y, z: Stereotaxic coordinates.

Random-effect analyses of 10 participants (*P* < 0.001, uncorrected for the entire search volumes).

**Figure 5:**
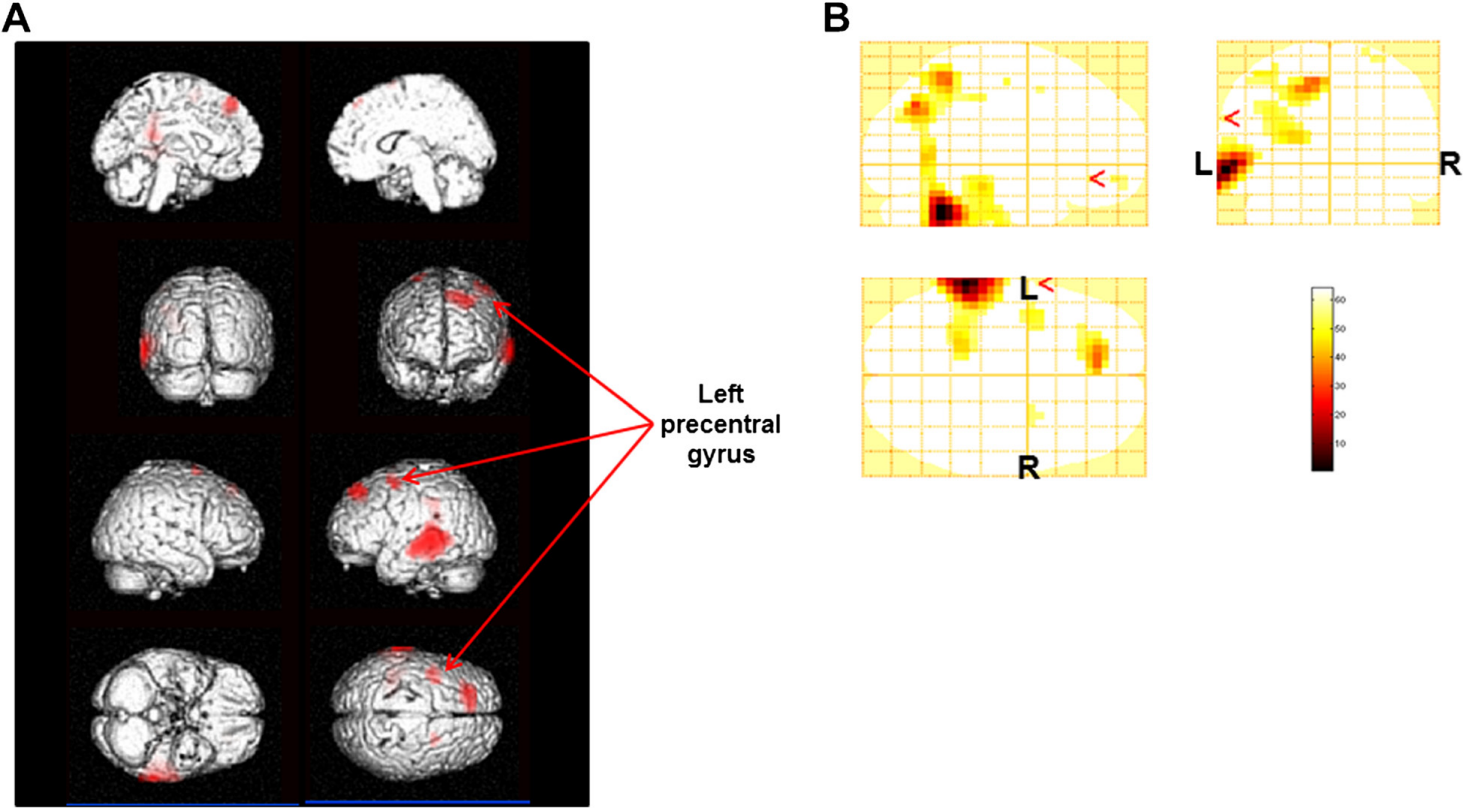
**Statistical parametric maps of beta power changes by 0-back test trials (random-effect analyses of 10 participants, P < 0.001, uncorrected for the entire search volumes).** Statistical parametric maps are superimposed on surface-rendered high-resolution MRI’s, and red indicates an increase of alpha power (**A**). Increase of alpha power (**B**) was shown on sagittal (upper left), coronal (upper right), and axial (lower left) sections. Color bar (lower right) indicates % increase of the beta power. R, right; L, left.

**Figure 6:**
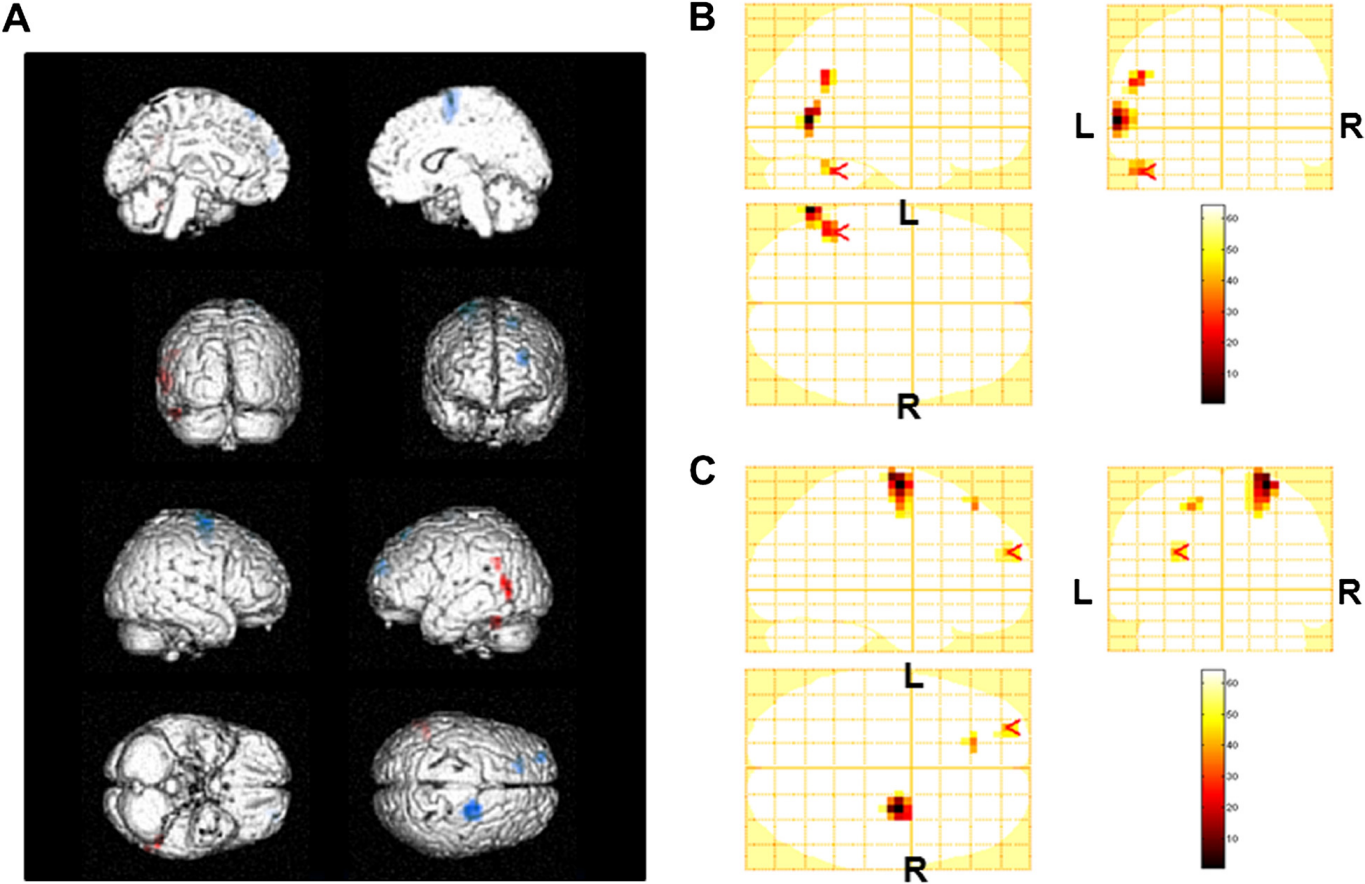
**Statistical parametric maps of beta power changes by 2-back test trials (random-effect analyses of 10 participants, P < 0.001, uncorrected for the entire search volumes).** Statistical parametric maps are superimposed on surface-rendered high-resolution MRI’s, and red indicates an increase of alpha power and blue indicates a decrease (**A**). Increase of alpha power (**B**) and decrease of alpha power (**C**) were shown on sagittal (upper left), coronal (upper right), and axial (lower left) sections. Color bar (lower right) indicates % increase (**B**) or % decrease (**C**) of the beta power. R, right; L, left.

**Figure 7:**
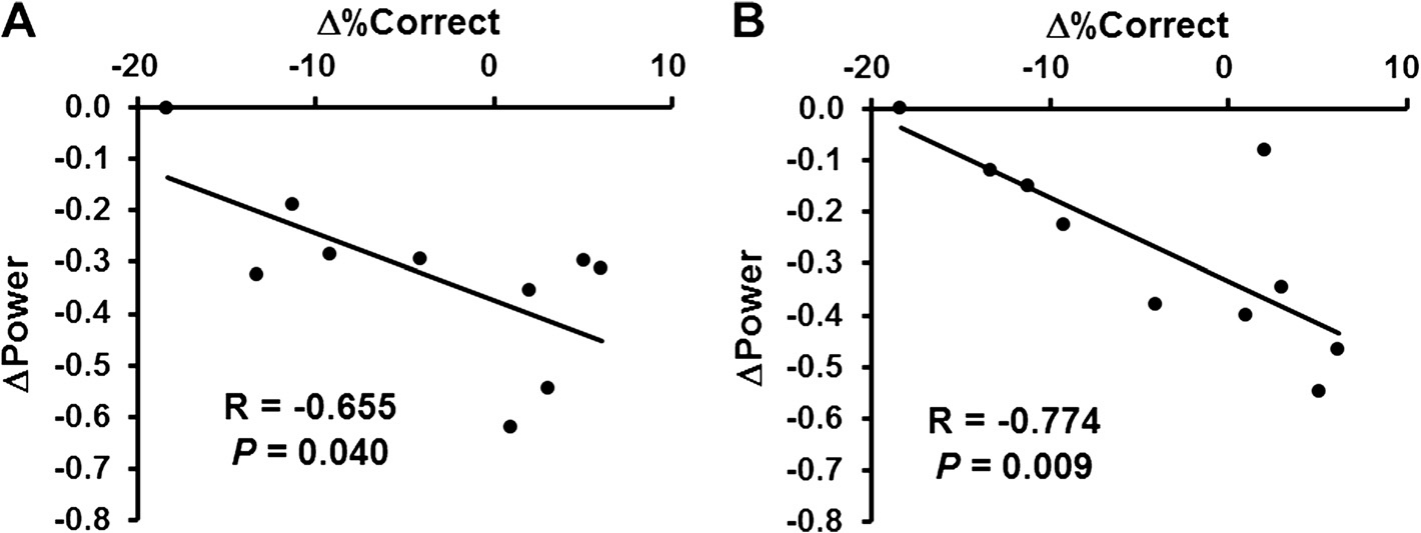
**Relationships between change of rate of correct performance [Δ%Correct] during the time course of the 2-back test trials and alpha power change (ΔPower) in the right middle frontal gyrus (Brodmann’s area 46) (A) and in the right superior frontal gyrus (Brodmann’s area 9) (B).** Linear regression lines, Pearson’s correlation coefficients, and P values are shown.

**Figure 8:**
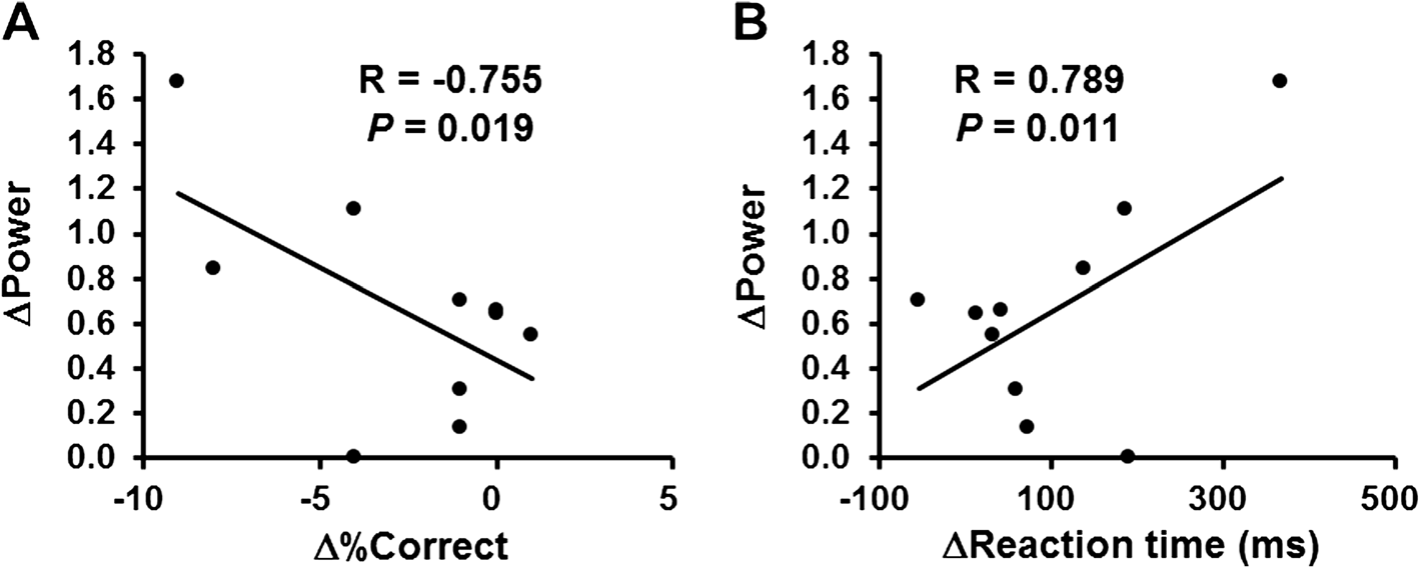
**Relationships between changes of rate of correct performance [Δ%Correct] (A) and reaction time (ΔReaction time) (B) during the time course of the 0-back test session and beta power change (ΔPower) in the left precentral gyrus (Brodmann’s areas 4, 6).** Linear regression lines, Pearson’s correlation coefficients, and P values are shown.

The alpha power changes in the right middle frontal gyrus (BA 46) and right superior frontal gyrus (BA 9) for the 2-back test session were lower than those for the 0-back test session. The beta power change in the left precentral gyrus for the 2-back test session was lower than that for the 0-back test session. In order to find the brain regions related specifically to task performance, we performed correlation analyses. The alpha power changes in the right middle frontal gyrus (BA 46) and right superior frontal gyrus (BA 9) were negatively associated with the change of percentage correct (Figures 5A and 6A, R = 0.655, P = 0.040 for the right middle frontal gyrus; Figures 5B and 6B, R = 0.774, P = 0.009 for the right superior frontal gyrus) for the 2-back test session. The beta power change in the left precentral gyrus (BA 4, 6) was negatively associated with the change in percentage correct (Figures 7 and 8, R = −0.755, P = 0.019) and positively associated with the change of reaction time (Figures 7 and 8, R = 0.789, P = 0.011) for the 0-back test session. We also performed the analyses within the delta and theta frequency bands. However, we could not identify the MEG results related to the task performance.

## Discussion

Oscillatory brain rhythms are considered to originate from synchronous synaptic activities of a large number of neurons [22]. Synchronization of neural networks may reflect integration of information processing, and such synchronization processes can be evaluated using MEG time-frequency analyses [23]. Aberrant resting-state oscillatory activities have been observed in various pathological conditions [15,24]. We found that, after the 0-back test, decreased alpha power was shown in the angular gyrus and increased levels were shown in the temporal, postcentral, and frontal gyrus. In contrast, after the 2-back test, decreased alpha power was shown in the frontal gyrus and increased levels were shown in the parietal lobule, parahippocampal gyrus, uncus, postcentral gyrus, and frontal gyrus. For beta power, after the 0-back test, increased power was shown in the temporal, frontal, cingulate, and precentral gyrus. After the 2-back test, decreased power was shown in the frontal gyrus and increased power was shown in the temporal gyrus and parietal lobule. In addition, the beta power change in the left precentral gyrus was negatively associated with task performance of the 0-back test session; and the alpha power changes in the right middle and superior frontal gyrus were negatively associated with the task performance of 2-back test session.

In our previous EEG study, beta power densities on the P3, Pz, and O1 electrodes and alpha power densities on the P3, Pz, O1, and O2 electrodes were decreased after a fatigue-inducing 2-back test session, while alpha power densities on the P3 and Pz electrodes were decreased after a fatigue-inducing 0-back test session. Although there are some similarities between the results of the EEG and MEG studies, the differences are apparent. EEG measures electrical fields, which are based on the difference in potentials between an EEG electrode and a reference electrode. In addition, the EEG signal is influenced by the electrical conductivity of intervening tissues such as the skull. In contrast, MEG measures magnetic fields, which does not use a reference, and MEG provides superior spatial resolution and signal-to-noise ratio relative to EEG. Therefore, MEG could identify accurate changes of the mental fatigue-related oscillatory brain activities that were not evident in our previous EEG study.

Multiple, broadly distributed, and continuously interacting dynamic neural networks are achievable through the synchronization of oscillations at particular time-frequency bands. Alpha, one of the large-scale rhythmic oscillations in the brain, is generated in the process of interactions between thalamocortical neurons and GABAergic (γ-aminobutyric acid) cells in the thalamic reticular nucleus [25,26]. This time-frequency band is related to complex cognitive processes such as attention, memory, and mental imagery [27-29]. Chaudhuri and Behan proposed that the fatigue is related to the activation of the thalamo-frontal feedback loops [5,28,30], and the overactivation of the thalamo-frontal feedback loops in order to compensate for the functional loss caused by fatigue has been reported in fatigue with some diseases such as multiple sclerosis [31-33] and chronic fatigue syndrome [34,35]. Since mental fatigue induced by 2-back trials suppressed spontaneous alpha power, i.e., desynchronization due to intrinsic events, in the frontal gyrus, this desyncronization might have some relationships with the activation of the thalamo-frontal feedback loops.

The motor cortex exhibits resting-state synchronization of the oscillations at beta frequency band [36,37]. It has been demonstrated that these oscillations appear to be under the direct control of GABAergic modulation [38,39]. These oscillations are facilitated by increasing the inhibitory drive of GABAergic interneurons via GABA-A receptors, which lengthens the inhibitory post-synaptic potential decay time, thereby reducing the frequency of the locally oscillating neuronal network population. Consequently, this serves to facilitate the recruitment of principal cells to the oscillating population, giving rise to an increase in the amplitude of the oscillatory power, as the participating neuronal pool is increased [40]. In contrast with the results of the 2-back test, the beta power in the left precentral gyrus was increased after 0-back test trials. Interestingly, the increased power in the left precentral gyrus was negatively associated with the percentage correct and positively associated with the reaction time of the 0-back test session. Inhibitory mechanisms apparently suppressed the beta power in the motor areas according to the level of impaired task performance, i.e., mental fatigue. Fatigue is a bio-alarm that senses risks, warns the organism, and orders rest. Therefore, some inhibitory mechanisms that order rest to in order to avoid overwork and even homeostatic catastrophe may exist, and we may recognize that the inhibition is caused by fatigue, resulting in a sensation of fatigue [41]. The increase in inhibitory input to the motor cortex with physical fatigue has been suggested in behavioral [42] and MEG [43] studies; enhanced and persistent activation of the inhibitory system may be related to the pathophysiology of chronic fatigue [44,45]. Since this inhibitory system alone causes impaired task performance or even cancellation of task trials, some compensatory mechanisms would be necessary to maintain the task performance. The 0-back test did not require a higher level of mental load; thus, compensatory mechanisms may not be necessary to maintain task performance during the task trials, overactivation of the inhibitory system seems to play a central role in the pathophysiology of mental fatigue caused by 0-back test trials, in contrast to the mental fatigue caused by 2-back test trials.

### Limitations

While the results of the present study are suggestive of the mechanisms of mental fatigue, only a limited number of participants and limited time-frequency bands were tested. To generalize our results, studies involving a larger number of participants and a variety of time-frequency bands will be needed. In addition, we did not measure the objective markers of mental fatigue in urine and serum [46] and did not investigate the relationship between the MEG data and these markers. Furthermore, although the participants did not complain of physical fatigue, we could not exclude the possibility that the MEG data was affected by physical fatigue. Finally, the use of the task with more burdens, such as 3-back or more, longer duration of tasks could effectively extract the fatigue as the difference of these two conditions. We used 30-min 2-back test as a fatigue-inducing task based on the results of our previous study [7]: To evaluate mental fatigue, participants completed advanced trail-making test (ATMT [11]) for 30 min, and after the 2-back test session for 30 min, ATMT performance was impaired, and this session was shown to be mental fatigue-inducing tasks. In the ATMT, circles numbered from 1 to 25 were randomly located on the display of a personal computer, and the participants were required to use a computer mouse to click the center of the circles in sequence, starting with circle number 1. The task performance was evaluated by number of errors. In addition, by using the 0-back and 2-back tasks, we could extract the fatigue as the difference related to the MEG responses to visual stimuli of these two conditions [47,48]. Therefore, we adopted 30-min 2-back test as a fatigue-inducing task.

## Conclusions

We identified mental fatigue-related changes in MEG spontaneous oscillatory activities. In particular, the beta power change in the left precentral gyrus (BA 4, 6) was negatively associated with the task performance for the 0-back test, and the alpha power changes in the right middle (BA 46) and superior (BA 9) frontal gyrus were negatively associated with task performance for the 2-back test. Two types of mental fatigue produced different types of the alterations of the oscillatory brain activities. We believe that our results contribute to providing new perspectives on the neural mechanisms underlying mental fatigue as well as developing evaluation methods and establishing a basis for treatment in order to overcome fatigue.

## Abbreviations

ANOVA: Analysis of variance; ATMT: Advanced trail making test; BA: Brodmann; BRAM: Brain Rhythmic Analysis for Magnetoencephalography; EEG: Electroencephalography; GABA: γ-aminobutyric acid; MEG: Magnetoencephalography; MNI: Montreal Neurological Institute; MRI: Magnetic resonance imaging; SPM: Statistical parametric mapping; VAS: Visual analogue scale.

## Competing interests

The authors declare that they have no competing interests.

## Authors’ contributions

MT took part in planning and designing the experiment, collected the data, performed the data analyses and drafted the manuscript. YS, AI, EK, and MF took part in planning and designing the experiment, collected the data, and performed the data analyses. YW took part in the planning and designing the experiment and helped drafting the manuscript. All authors read and approved the final manuscript.
